# Synthesis and Characterization of Amino Acid Decyl Esters as Early Membranes for the Origins of Life

**DOI:** 10.3390/membranes12090858

**Published:** 2022-08-31

**Authors:** Isabella Lago, Lissa Black, Maximillian Wilfinger, Sarah E. Maurer

**Affiliations:** Department of Chemistry and Biochemistry, Central Connecticut State University, 1615 Stanley St., New Britain, CT 06050, USA

**Keywords:** origins of life, vesicles, liposomes, abiogenesis, chemical evolution

## Abstract

Understanding how membrane forming amphiphiles are synthesized and aggregate in prebiotic settings is required for understanding the origins of life on Earth 4 billion years ago. Amino acids decyl esters were prepared by dehydration of decanol and amino acid as a model for a plausible prebiotic reaction at two temperatures. Fifteen amino acids were tested with a range of side chain chemistries to understand the role of amino acid identity on synthesis and membrane formation. Products were analyzed using LC-MS as well as microscopy. All amino acids tested produced decyl esters, and some of the products formed membranes when rehydrated in ultrapure water. One of the most abundant prebiotic amino acids, alanine, was remarkably easy to get to generate abundant, uniform membranes, indicating that this could be a selection mechanism for both amino acids and their amphiphilic derivatives.

## 1. Introduction

The synthesis of amphiphiles on early Earth 4 billion years ago has previously been proposed through atmospheric, interstellar, and geological processes [1,2,3,4]. These molecules are hypothesized to be essential to the origins of life, serving as containers for metabolic and genetic components of early cells [5]. These molecules are also useful as they can self-assemble into colloids and perhaps concentrate from solution through aggregation behaviors, unlike the water-soluble biomolecules [6,7].

Prebiotic chemical synthesis has long been proposed to generate the molecules needed for metabolic and informational molecules of life; for recent examples, see [8,9,10,11]. Specifically, spark discharge reactions and meteorite extracts often contain amino acids [12,13,14]. The amino acids produced in these high energy reactions are not the biological set of twenty amino acids, but rather a modified alphabet which includes some biological amino acids (e.g., glycine, alanine, serine, glutamic acid) and some nonbiological amino acids (e.g., β-alanine, isoserine, aminobutyric acid). Amino acids are classified biochemically by the sidechain structure, and amino acids with smaller nonpolar side chains are generally more abundant from prebiotic sources than the larger, polar side chains. Additionally, the positively charged amino acid sidechains are largely missing from these prebiotic alphabets. These amino acids are usually speculated as evidence of a path to proteins, however, here, we propose that they could also be used to generate prebiotic vesicles.

While much of the focus on prebiotic vesicle formation to date has been on medium and long chain carboxylic acids (i.e., “fatty acids”) [15,16,17], the production of a variety of amphiphiles from these abiotic syntheses is much more likely. In fact, from Fischer–Tropsch-like reactions, alcohols may have been the most abundant hydrocarbon produced [18]. Additionally, fatty carboxylates, the deprotonated form of the carboxylic acid, precipitate in the presence of divalent cations [19], and while this is easily overcome using mixtures of amphiphiles or keeping the pH below the pKa [20,21,22,23,24], it seems unlikely that the highly researched pure fatty acid membranes were as relevant to abiogenesis on Earth as originally thought.

Even if medium and long chain alcohols were more abundant than other amphiphilic materials, their hydroxyl head group is not sufficiently polar to allow for membrane formation on its own. These alcohols form oil droplets, unless mixed with a more polar molecule, such as a fatty carboxylate or alkyl ammonium [15,22]. There are also a number of ways to modify both fatty acids and fatty alcohols through prebiotic synthesis to generate more stable amphiphiles. Over 40 years ago, both mono- and diacyl glycerols from fatty acids, alcohols, and aldehydes were shown to form from dry down reactions [16]. In more recent work, dry down reactions have been shown to form a variety of amphiphiles including: decyl phosphates from the condensation of alcohols and phosphate [25], N-decyl amides from fatty acid and amino acids [26], and phosphatic acid from glycerol, diamidophosphate (DAP), and nonanoic acid [27]. It is becoming apparent that fatty acids were just one among many prebiotically available amphiphiles. This mixed membrane composition also mirrors modern cell membranes that are composed of lipids with a variety of head group chemistries and tail lengths/unsaturation, which, while highly regulated, supports the usage of mixed membranes at the origins of life.

Perhaps surprisingly, pure systems of amphiphiles have been shown to be less stable than mixtures against environmental conditions of temperature, pH, salinity, and dissolved solutes [15,19,20,21,22]. Fortunately, mixtures of these molecules would have been difficult to avoid in uncontrolled geological settings. It has been shown repeatedly that mixtures that contain alcohols and a more polar or charged head group are stable to divalent cations, temperatures in excess of 60 °C, high sea salt concentrations, pH as low as 2 and as high as 12, and the addition of organic molecules.

**Scheme 1:**
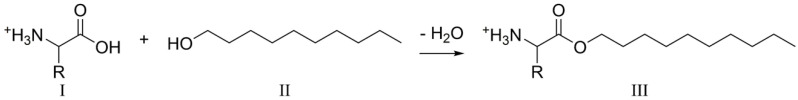
Condensation of amino acid (I) with decanol (II) to form of amino acid decyl esters (III). R represents the amino acid side chain.

One of the head group chemistries that has not yet been identified as prebiotically plausible in the literature is positively charged amine head groups. Both choline and ethanol amine are abundant in modern cell membranes, both of which contain a positively charged nitrogen. The addition of positively charged amphiphiles to our prebiotic chemical inventory could enhance the functionality and stability of the first cell membranes. This work reports the formation of such amphiphiles from a prebiotically plausible dehydration reaction of medium chain alcohols with amino acids, both of which are abundant from proposed prebiotic synthesis (Scheme 1). By looking at reaction conditions which lead to amphiphiles and the sets of amino acid decyl esters that easily form membranes, we believe this type of amphiphile and its spontaneous assembly into vesicles would have been likely on early Earth.

## 2. Materials and Methods

### 2.1. Materials

DL-leucine and decyl alcohol were obtained from Acros Organics (98+% Fair Lawn, NJ, USA). L-methionine, L-lysine, and D-aspartic acid were obtained from Sigma Chemical Co. (St. Louis, MO, USA). DL-tryptophan, L-arginine, and DL-serine were obtained from Eastman Organic Chemicals (New York, NY, USA). L-phenylalanine was obtained from Sigma-Aldrich (St. Louis, MO, USA). DL-alanine, L-glutamic acid, hydrochloric acid, chloroform, acetonitrile, and L-tyrosine were obtained from Fisher Scientific Company (Fair Lawn, NJ, USA). L-proline and L-valine were obtained from Tokyo Chemical Industry (Oregon, USA). L-cysteine was obtained from Alfa Aesar (Heysham, England). L-histidine, ethanol, and methanol were obtained from Aldrich Chemical Co. (Milwaukee, WI, USA).

### 2.2. Prebiotic Ester Synthesis

In individual glass test tubes, 1 mmol ± 10% of each amino acid, 1 mmol of decanol, and 5 mL of 0.5 M HCl in 50:50 ethanol/water was added. The tubes were each vortexed for 30 s and placed into an oven at either 60 or 80 °C for 3–7 days. The samples were rehydrated with 2–5 mL of ultrapure water (18.2 MΩ; Millipore, Burlington, MA, USA). 

Products were purified using chloroform extraction. In disposable borosilicate culture tubes, 1 mL of rehydrated reaction mixture was mixed with 1 mL of methanol, then vortexed for 30 s. Using a gas tight Hamilton syringe, 2 mL of chloroform was added to each tube and mixed. Tubes were centrifuged using a Centricone centrifuge for 10 min at 1500 rpm. The chloroform was transferred to preweighed tubes using a glass pipette. The extracts were left to air dry at room temperature and the final mass was determined. Theoretical yield was determined as the mass of 100% product from Scheme 1.

### 2.3. Vesicle Analysis

Both purified and nonpurified materials were used in the characterizations. Macroscopic observations were characterized based on turbidity, time for phase separation to occur, and the presence or absence of precipitates. Samples were then stained with 100 µM Nile Red dye and examined with fluorescence microscopy for the presence or absence of vesicles and other structures. Vesicles were generally defined as a dark boundary with a lighter interior. “Oil” and “oil droplet” (sometimes called “oil-in-water emulsion”) was identified as a bright dark round spot. “None” indicated no fluorescent structures were observed. The resolution was about 400 nm; anything smaller (like a micelle) would not be observed.

Samples were diluted to a final concentration of 70% acetonitrile (Optima grade, FisherScientific, Hampton, NH, USA), and then analyzed using LC-MS (Ultimate 3000 UHPLC, VelosPro MS, ThermoScientific, USA). An Accucore HILIC-Z column with a 30% water, 70% acetonitrile (FisherScientific, Waltham, MA, USA, Optima Grade) isocratic elution was used for separation. MS detection used atmospheric pressure chemical ionization (APCI; 5 kV spray, 275 °C capillary temperature, 60% S-Lense RF level).

## 3. Results

Amino acid decyl esters were characterized for structure formation and yields. The synthesis was performed at both 80 and 60 °C to test the effect of temperature on the reaction. Samples were analyzed through macroscopic characterization, fluorescence microscopy and liquid chromatography–mass spectrometry (LC-MS).

Initially, samples were examined for structure-formation macroscopically, looking at the turbidity (Figure S1). The samples that formed stable emulsions appeared cloudy or turbid (Table S2); however, many of these emulsions were not stable and phase-separated over time. There were a few differences between the 60 and 80 °C samples even in the macroscopic observations. At 80 °C many of the samples were notably brown (Figure S1). At 60 °C, browning only occurred slightly in the tryptophan sample (Figure S2).

**Figure 1:**
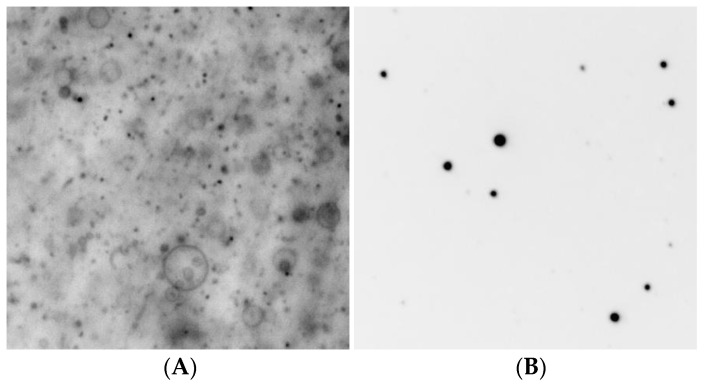
Micrographs of rehydrated products. (**A**) Cysteine decyl esters form poly disperse membranes. (**B**) Histidine decyl esters form oil droplets. Micrograph size = 100 µM.

Samples were also examined using fluorescence microscopy. Vesicles were identified as having a bright circular exterior with a lighter aqueous interior (Figure 1A and Figure S3). The microscopic analysis revealed that many of the samples did form membranes, as indicated in Table 1. All of the aliphatic side chain amino acid esters (Gly, Pro, Leu, Ala, Ser, Val) and the acidic side chains (Asp, Glu, Cys) formed vesicles. The amines (His, Arg, Lys) and the aromatics (Tyr, Trp, Phe) did not have membrane-forming products. Some of the samples had oil droplet emulsions as the main microscopic structures (Figure 1B and Figure S4). Samples dehydrated at 60 °C with high turbidity were further diluted (4×) with water, and vesicles were then able to form, as indicated by an asterisk in Table 1.

Of special note, alanine, glutamic acid, aspartic acid, and cysteine formed copious amounts of membranes with ease. We would like to emphasize, here, that these samples were simply mixtures of amino acids and decanol dried under acidic conditions for 3–8 days. The effortlessness of forming vesicles with this method cannot be overstated and was really quite extraordinary, lending to its ability to function as a prebiotic synthesis.

These vesicle-forming products were relatively stable as membranes at room temperature for the duration of the experiments (months). Additionally, adjusting the pH of the samples to around 7 did not significantly change the morphology of the structures. At pH 10 and greater, esters are known to quickly hydrolyze (<24 h), so further pH adjustment was not attempted. Preliminary assessment of the critical vesicle concentration (CVC) using turbidity at 550 nm indicates values of around 10–20 mM for Ala, Asp, Cys, and Ser, which is similar to the CVC of decanoic acid [28].

The percent yield was determined by chloroform extraction with the expectation that hydrophobic products would preferentially partition into the chloroform phase (Table 1). Yields between 40 and 70% occurred with most products at 60 °C, which has been observed in similar reactions [16,25,27]. Notably the positively charged side chains (His, Lys, Arg) had very low yields. These low yields in part may be due to lower solubility in the chloroform phase, because of the double ammonium headgroup and, indeed, the histidine ester product was noted in the water phase by LC-MS after purification. There does not seem to be a trend with the other amino acid side chain chemistries and yields.

For all samples, the mass associated with the [M + H]^+^ ion was detected, generally as the base peak, indicating that decyl ester formed in all amino acids tested. Peptide products from the condensation of two or more amino acids, which can also form under these conditions, were not detected in the rehydrated reaction mixture using this analysis method.

**Figure 2:**
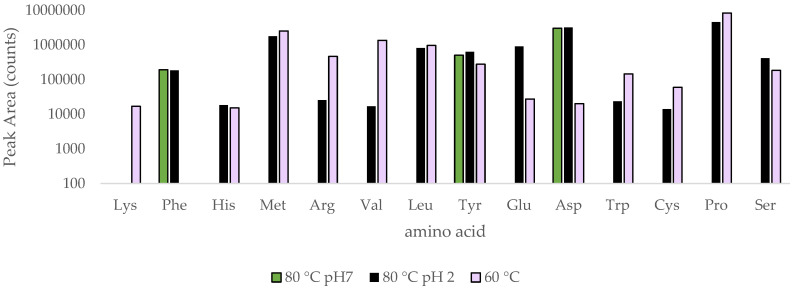
MS peak area of each amino acid decyl ester synthesized under 3 different conditions. Peak areas were determined at the expected mass for the product of each amino acid. Green bars were dried with a starting pH of 7 at 80 °C; black bars at a starting pH of 2 at 80 °C; pink bars at a starting pH of 2 at 60 °C. Note that the *y*-axis is logarithmic and starts at 100. Comparisons within amino acids are possible; however, due to lack of standards and different ionization rates, comparisons between amino acids are not overly valuable. If no bar is present, the products were not present/below the limit of detection. While *n* = 1, the error of a triplicate alanine ethyl ester sample was within 10%.

The MS data were also used to assess the amount of product formed under three dehydration conditions: 80 °C, pH 2; 80 °C, pH 7; and 60 °C, pH 2 by evaluating the peak area of the exact mass of the predicted product (Figure 2). While peak areas can be compared for a single decyl ester, comparisons of peak areas between different amino acids cannot be made, as each amino acid differs in its ability to ionize, and standards for these products are not commercially available.

Reactions at pH 7 were mostly unable to esterify; products were only found with phenylalanine, tyrosine, and aspartic acid. Both phenylalanine and tyrosine have aromatic sidechains. The aspartic acid may react on both the side chain and the c-terminus of the amino acid; although, notably, glutamate did not form products under these conditions. At pH 2, samples mostly showed similar product peaks regardless of temperature (Ser, Pro, Leu, Tyr, Met). The acidic sidechains seemed to strongly favor higher temperatures (Glu and Asp), while other amino acids had larger peak areas at lower temperatures (Arg, Val, Trp, Cys). The mass of the lysine product was not detected at 80 °C; phenylalanine did not have any product mass signal at 60 °C. This result suggests that environmental selection could be used to drive the production of specific amphiphiles from a starting mixture of amino acids.

## 4. Discussion

The formation of positively charged amphiphiles from a prebiotically plausible synthesis presents a novel chemical functionality for generating membranes for the origins of life. The products formed here demonstrate that a wide range of amino acids can be used to generate these products. We expect that other amino acids, including nonbiological amino acids, would also form these amphiphile products. We also did not control for the chirality of the amino acids. Some of the amino acids were racemic and others were L-form. We did a brief test with D- and L-alanine, and the sample looked identical to both DL- and L-products morphologically. However, there may be unique properties of the membranes that are related to the chirality of the amino acids, which we hope to explore further in the future.

Interestingly, we did not detect the ethyl ester product, even though ethanol was used as the solvent in the reaction. We suspect this is due to the ethanol (bp 78 °C) evaporating at a faster rate than both water (bp 100 °C) and decanol (bp 230 °C). This provides an interesting selection mechanism for the longer chain products. We suspect that in some of the samples, unreacted decanol remained and was acting as a membrane stabilizer. Decanol does not form membranes on its own but is well-known to form membranes in mixtures [15,19,20,22]. Some of the amino acid products did not form vesicles or had abundant oil droplets concurrent with vesicles. It is likely that modifying the environment or adding a different cosurfactant could produce membranes. We additionally hope to expand this work in the future focusing on a larger diversity of starting materials and the relationship between the dehydration environment and amphiphile formation and stability.

The results also suggest that environmental conditions could determine which products formed based on temperature and pH of the solutions. Low pH conditions are often found from geothermal activity, which would have been more prevalent on a young planet. Higher temperatures were also likely as the Earth cooled from the moon-forming impact and internal heating. Geothermal pools on volcanic islands could have provided the ideal location for a low pH environment that dried prebiotic molecules, leading to a diversity of amphiphiles self-assembling into the first cell membranes. One of those amphiphiles was likely the amino acid esters, providing a positively charged headgroup to increase the functionality and stability of early cells.

## Figures and Tables

**Table 1:** Summary of product characterization for each amino acid.

Amino Acid	3-Letter Abbreviation	Yield 60 °C	Microscopic Structures 60 °C	Microscopic Structures 80 °C
Methionine	Met	70	Amorphous solids	None
Aspartic Acid	Asp	68	Vesicles *	Vesicles
Glycine	Gly	65	Vesicles	Oil
Proline	Pro	62	Vesicles	None
Leucine	Leu	58	Vesicles *	Vesicles *
Tryptophan	Trp	57	Crystals	Crystals
Alanine	Ala	51	Vesicles *	Vesicles
Cysteine	Cys	47	Vesicles *	Vesicles
Glutamic Acid	Glu	45	Vesicles *	Vesicles
Serine	Ser	41	Oil, vesicles	Vesicles
Valine	Val	39	Vesicles *	Vesicles
Tyrosine	Tyr	38	Crystals	Crystals
Arginine	Arg	12	Oil	Oil
Histidine	His	3	None	Oil
Lysine	Lys	3	None	Oil
Phenylalanine	Phe	2	Oil	Crystals

* Indicates that samples needed to be diluted 4× for vesicles to be seen.

## Data Availability

Data is available through the supplemental materials provided.

## Supplementary Materials

The following supporting information can be downloaded at: https://www.mdpi.com/article/10.3390/membranes12090858/s1, Figure S1: Macroscopic characterization of decyl esters at 80 °C; Figure S2 Macroscopic characterization of decyl esters at 60 °C; Table S1: Macroscopic characterization of decyl esters from various amino acids; Figure S3: Micrographs of amino acid decyl esters that formed vesicles; Figure S4: Micrographs of amino acid decyl ester aggregates.
