# Supplementary material for: Synthesis and Characterization of Amino Acid Decyl Esters as Early Membranes for the Origins of Life
Source: Membranes (Basel). 2022 Aug 31;12(9):858. doi: 10.3390/membranes12090858 (PMC9502762; doi:10.3390/membranes12090858)
Supplement: Supplementary file 1 [file membranes-12-00858-s001.zip › membranes-1877028-supplementary.pdf]

membranes

Supplemental Materials

Synthesis and Characterization of Amino Acid Decyl Esters as Early Membranes for the Origins of Life

Isabella Lago, Lissa Black, Maximillian Wilfinger and Sarah E. Maurer *

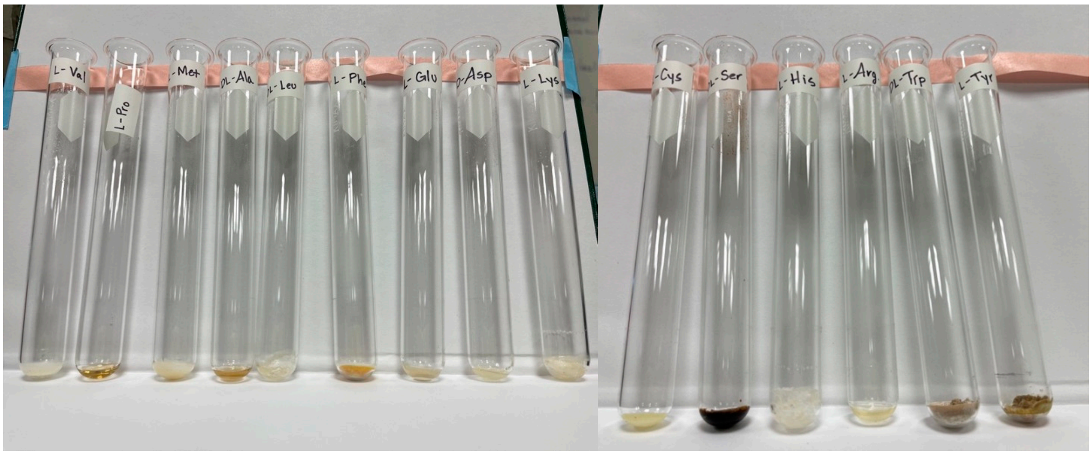

Figure S1. Macroscopic characterization of decyl esters from amino acids dried at 80 °C for 5 days before rehydration. Note that many of the samples were brown, indicating side reactions occurring.

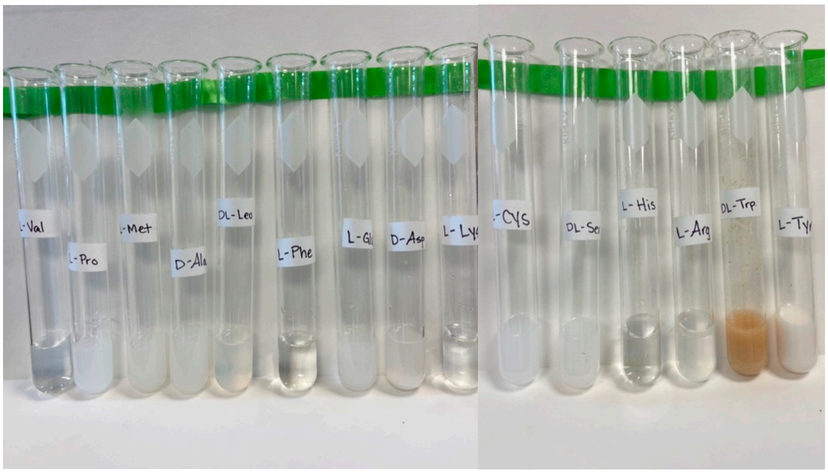

Figure S2. Macroscopic characterization of decyl esters from amino acids dried at 60 °C for 7 days after rehydration. Note that many of the samples are turbid indicating colloid formation. Tryptophan was the only sample at 60 °C that was noticeably brown.

Samples were observed for turbidity indicating aggregates (Figure S1-S2). Varying degrees of turbidity were found in these samples corresponding to microscopic structures of vesicles, oil droplets and crystalline structures (see Table 1, main text).

Table S1. Macroscopic characterization of decyl esters from various amino acids. "X" indicates sample was turbid. "~" indicates sample was slightly turbid, but large presence of particulates may have contributed to macroscopic appearance.

Amino Acid:	Turbid at 80°C	Turbid at 60°C
Alanine	X	X
Arginine	X	X
Aspartic Acid	X	X
Cysteine	X	X
Glutamic Acid	X	X
Histidine	X	
Leucine	X	X
Lysine	X	
Methionine		X
Phenylalanine	~	
Proline		X
Serine	X	X
Tryptophan	~	X
Tyrosine	~	X
Valine	X	

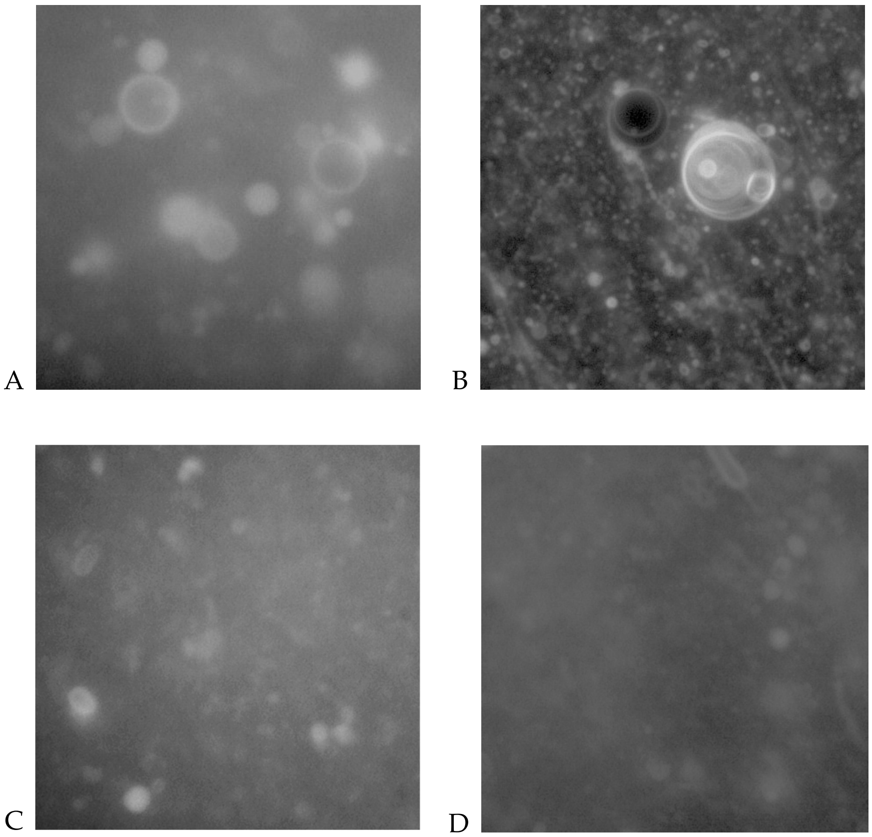
A B C D

Figure S3. Micrographs of amino acid decyl esters that formed vesicles. A) alanine; B) aspartic acid; C) proline; D) valine

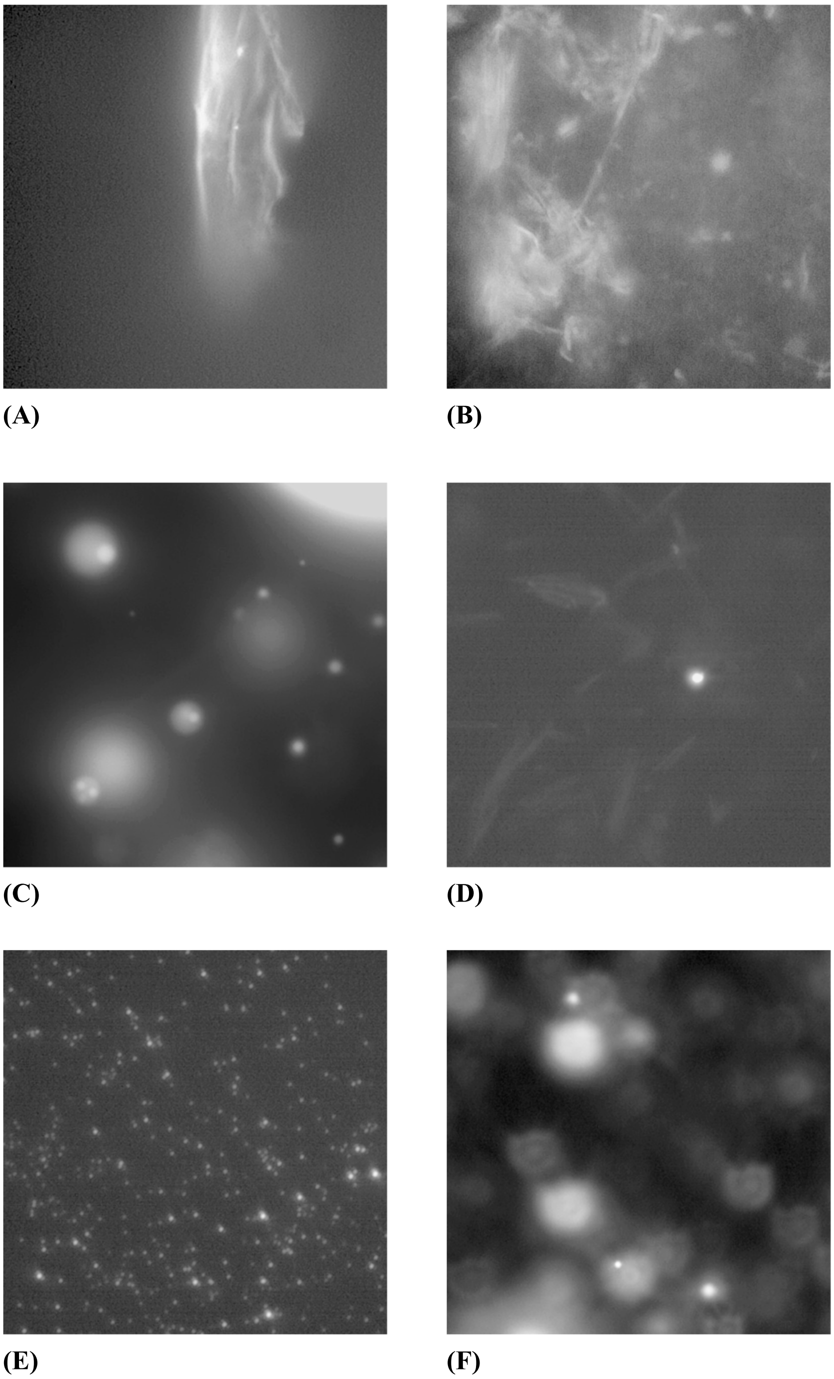

Figure S4. Micrographs of amino acid decyl ester aggregates. A) methionine; B) Tyrosine; C) Serine; D) Tryptophan; E) Histidine; F) Lysine
